# Digital finance development in China: A scientometric review

**DOI:** 10.1016/j.heliyon.2024.e36107

**Published:** 2024-08-10

**Authors:** Qiwei Li, Xinyu Zhang

**Affiliations:** a Faculty of Economics, RUDN University, Moscow, Russia; b Institute of Foreign Languages, RUDN University, Moscow, Russia

**Keywords:** China, Digital finance, Digital currency, Digital inclusive finance, Fintech, Blockchain technology, Scientometric analysis, Content analysis

## Abstract

The continuous integration of digital technology and finance has spurred the rapid development of the digital finance industry, making it a critical area of interest for scholars. This study combines quantitative research methods using Citespace software for scientometric analysis and qualitative research methods involving manual selection and content analysis of key literature to summarise the research status, hot topics, and frontiers in the field of digital finance in China. The research findings highlight several influential factors in the digital finance literature, such as regional and journal distribution, institutional and author collaboration, and highly cited literature. Currently, the four most important and cutting-edge research areas are digital currency, digital inclusive finance, fintech and blockchain technology. Furthermore, an analysis of the development trends in digital finance research is conducted and future research perspectives are suggested.

## 1 Introduction

The advent of the digital economy has emerged as a pivotal force propelling global economic expansion, representing a primary arena for competitive dynamics. Particularly underscored by the onset of the COVID-19 pandemic, the imperative of digitalizing economies has surged to the forefront of global priorities. Within the expansive realm of economics, finance stands as a cornerstone, wielding a decisive and catalytic influence in steering the trajectory of the digital economy through the avenue of digital finance [1]. Characterized as an emergent nexus intertwining financial principles with digital technologies, digital finance continually undergoes refinement and expansion in both its scope and substance.

In its nascent phases, digital finance predominantly accentuated technological advancements within financial products and operational paradigms. Presently, this domain has matured to encompass a multifaceted spectrum of technological innovations spanning the entire breadth of the financial landscape, encompassing facets such as financing mechanisms, investment strategies, lending frameworks, currency dynamics, payment modalities, consultancy services, and beyond [2]. However, amidst this burgeoning landscape, a coherent and universally accepted definition of digital finance is still elusive, reflecting the evolving nature of this interdisciplinary domain.

The convergence of finance and technology has deep historical roots, evidenced by its enduring presence within international journal literature and governmental documentation. Within these spheres, terminologies such as "financial technology," "digital finance," or "electronic finance" are recurrently employed. In the Chinese context, however, terminology preferences diverge slightly, with phrases like "digital finance," "financial technology," or "internet finance" prevailing.

The Irish International Digital Research Centre [3] elucidates fintech as the amalgamation of technological innovation and digitization within financial services. Concurrently, the Financial Stability Board [4] delineates fintech as encompassing technologically driven innovations in financial services, potentially engendering novel business models, applications, processes, or products that significantly impact financial markets, institutions, and service provision.

Similarly, the European Commission's Directorate-General for Financial Stability, Financial Services, and Capital Markets Union [5] characterizes digital finance as the transformative influence of modern technologies on the financial services sector, encapsulating a myriad of products, applications, processes, and business models that reshape conventional banking and financial service delivery paradigms.

Moreover, the People's Bank of China et al. [6] define internet finance as a novel financial operational model leveraging internet technology and information communication technology, wherein traditional financial institutions and internet entities collaborate to facilitate fund transfers, payments, investments, and intermediary services. Huang & Huang [7] offer nuanced perspectives, contending that "internet finance" primarily denotes the involvement of internet corporations in financial service provision, emphasizing the technological dimension, whereas "financial technology" accentuates technological attributes. In contrast, "digital finance" assumes a more encompassing and neutral stance, embodying a broader spectrum of financial technological integration.

Wang & Hu [8] offer a comprehensive synthesis of extant literature concerning the definition of digital finance. They posit that digital finance embodies the digital transformation of financial services and currency, facilitated by the integration of advanced digital technologies such as big data analytics, cloud computing, blockchain, and artificial intelligence. This transformation is spearheaded by a collaborative effort among traditional financial institutions, internet enterprises, and fintech entities. Recognizing the absence of a universally accepted standard for defining digital finance across diverse scholarly works, the article adopts the definitions articulated by Huang & Huang and Wang & Hu [7,8]. This inclusive approach encompasses terminology such as digital finance, fintech, and internet finance under the overarching umbrella term of digital finance.

Digital finance stands as a transformative innovation stemming from the profound amalgamation of financial principles and technological advancements, representing a synergistic evolution at the nexus of finance and technology. In the context of China's developmental trajectory, digital finance, while initiating later, has exhibited remarkable strides in its progression. The establishment of the "Digital Inclusive Finance Index" of China by the Peking University Digital Finance Research Centre [9], spanning the period from 2011 to 2020, unveils a trajectory characterized by sustained and rapid expansion within the realm of digital finance.

A nuanced examination of sub-indicators reveals a noteworthy trend wherein China's digitization level has surpassed the overall advancement of digital inclusive finance, indicative of the rapid assimilation of digital finance solutions by users, propelled by its inherent attributes of mobility, cost-effectiveness, and user convenience. As the breadth of digital finance services broadens and the depth of digital support intensifies, the utilization of digital finance extends across diverse domains encompassing payments, credit facilities, insurance provisions, and investment mechanisms [2].

Presently, China assumes a pivotal role in the global landscape of digital finance, as underscored by the "IDC Global FinTech Rankings" released by the esteemed research firm IDC Financial Insights. Notably, 14 Chinese enterprises secured positions within the roster of the top 100 global fintech companies in 2022, emblematic of the accelerating tempo of technological innovation and digital metamorphosis pervading the nation's financial sector.

The burgeoning landscape of digital finance has attracted considerable scholarly attention both domestically in China and internationally, prompting a proliferation of exploratory investigations into this evolving domain. Most of the literature on digital finance emerged after 2015, with the majority focusing on empirical research on specific topics within the field of digital finance. For example, studies by Corbet et al. [10] and Conlon & McGee [11] found that cryptocurrencies cannot serve as effective hedges or safe havens during economic shocks such as COVID-19. Kou et al. [12] evaluated fintech-based investments in European banking services, identifying the most significant fintech-based investment alternatives to improve the financial performance of European banks. Li et al. [13] examined the impact of digital inclusive finance on household consumption in China and explored the mechanisms of its effect. Additionally, in literature focusing on China, much attention is given to the impact of digital finance on the green economy. For instance, Zhou et al. [14] studied the role of fintech and green finance in promoting green economic growth in China, while Wang et al. [15] analyzed the impact of digital inclusive finance on China's carbon emissions.

In recent years, numerous review articles have analyzed the existing literature on digital finance [2,16,17]. However, the dynamic and nascent nature of digital finance, coupled with its relatively new conceptualization, has resulted in a fragmented research landscape characterized by a lack of standardized frameworks. This is specifically evidenced by limitations in comprehensive literature searches due to topic selection and small sample sizes due to methodological constraints. Additionally, in China, the scale and transformative impact of fintech or digital finance is unparalleled. Previous studies have rarely provided a systematic synthesis of research in the field of digital finance in China.

In this context, this paper aims to systematically review the literature on digital finance in China since 2013, thereby addressing this gap. The study seeks to elucidate the trajectory of research progress through visual analysis, distill prevalent themes and frontier inquiries, elucidate the evolutionary path of digital finance in China, identify key avenues for development and academic exploration, and highlight intrinsic deficiencies and urgent challenges that merit attention from the academic community. Specifically, this paper contributes to the current research in the following aspects.

Firstly, this paper precisely defines the concept of digital finance and summarizes the scope of themes it encompasses. Digital finance is an emerging field that combines digital technology and financial services, utilizing technologies such as the Internet, big data, blockchain, cloud computing, and artificial intelligence to provide financial services at lower costs and higher efficiency, thereby extending the reach of financial services. Digital finance is driven by traditional financial institutions, internet companies, and fintech firms, facilitating the transformation and innovation of financial services, enhancing financial inclusion and service efficiency. It not only encompasses improvements to traditional financial services but also includes a range of new financial products and services, such as mobile payments, internet lending, and digital currencies.

Secondly, this paper offers a more comprehensive literature search, covering a broader range of research topics within digital finance. Based on our definition of digital finance, the covered research topics include digital inclusive finance, fintech, internet finance, digital currency, cryptocurrency, peer-to-peer lending, crowdfunding, mobile banking, mobile payments, insurtech, robo-advisors, and related derivative themes. This comprehensive survey provides a panoramic view of research in the field of digital finance in China and offers guidance for future studies on the global digital finance landscape.

Thirdly, we provide a perspective on digital finance from the Chinese context. Compared to developed countries, China's digital finance industry has many unique characteristics. China leads in areas such as mobile payments, online lending, digital insurance, and online investments, while it lags relatively in cryptocurrencies and cross-border payments. Although China has a large number of digital finance companies, the market is primarily dominated by major tech companies, particularly the four internet giants known as “BATJ” (Baidu, Alibaba, Tencent, and JD.com). Furthermore, the development of digital finance in China has significantly promoted financial inclusion, providing low-cost and convenient financial services, including payments, billing, and deposits and loans, to small and micro enterprises and low-income populations in underdeveloped and remote areas. This digital finance revolution holds global significance due to its notable contributions to promoting financial inclusion.

Through this comprehensive undertaking, the paper aims to furnish stakeholders with a holistic comprehension of the research landscape within digital finance, thereby furnishing valuable insights into prospective developmental trajectories within this burgeoning field. This study attempts to answer the following research questions (RQs).

RQ1 What are the main areas of research in digital finance?

RQ2 In China, which institutions, journals, and authors are leading in the field of digital finance?

RQ3 What are the most influential articles and themes in China, and what are the main research frontiers in the Chinese digital finance literature?

RQ4 What are the future research directions?

The following sections are organized as follows: Section 2 delineates the research methodology and data sources used in this study. Following this, Section 3 conducts a bibliometric analysis, while Section 4 provides a content analysis. Section 5 discusses results and presents future research directions. Section 6 presents the limitations of this work and concludes.

## 2 Methodology and data sources

### 2.1 Methodology

Scientometrics, as a scholarly discipline, concerns itself with the quantitative measurement and analysis of academic literature. Fundamental research inquiries within this domain encompass the assessment of the impact of research papers and academic journals, the comprehension of scholarly citations, and the utilization of such metrics in policy formulation and management contexts [18]. Employing scientometric analysis facilitates the elucidation of the historical trajectory, developmental pathways, and emergent frontiers within a specific research field, thereby elucidating the establishment and consolidation of novel disciplines [19].

This analytical approach empowers scholars to delve into the nuances of a particular research domain, discern prevailing trends, delineate regional and source distributions, scrutinize citations and co-citations, and ultimately derive actionable insights. By furnishing researchers with a robust framework for evaluating extant scholarship within a given field, scientometric analysis aids in delineating the knowledge structure and its evolutionary trajectory. Moreover, it enables the identification of distinct subtopics and their conceptual frameworks, thereby unveiling research frontiers and hotspots within the discipline.

Citespace, developed by Dr. Chaomei Chen, is the preferred scientometric software for this study and serves as a powerful tool for conducting such analyses. It facilitates the identification of scientific literature and the visualization of development trends and contemporary dynamics within research domains [20]. Central to its functionality is the provision of insights into emerging trends within knowledge domains, thereby affording researchers invaluable insights into the evolving landscape of scholarship.

This paper adopts a mixed-method approach that combines scientometric and content analysis to conduct both quantitative and qualitative analyses of research related to digital finance. First, we utilize the scientometric analysis method facilitated by Citespace software to comprehensively examine time trends, source distribution, core authors, and other significant characteristics within the field of digital finance research. This analysis helps identify the historical evolution of research themes and noteworthy pivotal topics. Additionally, it has the advantage of handling large volumes of literature data, providing a comprehensive summary of the 5204 literatures involved in this study.

To identify the research frontiers in the field of digital finance, we complement the scientometric analysis with qualitative research methods. While scientometric methods can be combined with content analysis, unlike scientometric methods, content analysis is a qualitative approach aimed at providing a more in-depth understanding of the literature. For example, when conducting citation analysis, we use the total number of citations as an indicator of an article's importance and influence, and select a subset of highly cited articles from different time spans for detailed content analysis. Through meticulous content analysis of the literature, this paper elucidates popular hot topics and major research areas, thereby providing a comprehensive literature review.

The rapid development of digital finance in China has led to an abundance of related research, making it increasingly difficult to track these studies using traditional literature review methods. Systematic literature reviews typically condense a large number of documents into a smaller, significant subset for analysis. In contrast, scientometric analysis aims to cover the research field as comprehensively as possible [20]. This paper strives to provide a thorough overview of the digital finance research field, and after retrieving 5204 documents through literature searches, no further reduction of the dataset is performed during the scientometric analysis phase. Therefore, the mixed-method approach of combining scientometric methods and content analysis is the optimal choice for this study, as the combination of quantitative and qualitative methods helps the authors achieve comprehensive results. Moreover, the mixed-method approach facilitates a rigorous and coherent analysis of the research literature.

### 2.2 Data sources

The sample data utilized in this study originates from the CSSCI (Chinese Social Sciences Citation Index) core journals available within the CNKI (China National Knowledge Infrastructure) database. The timeframe selected for data collection spans from January 1, 2013, to December 31, 2022. The choice of commencing the analysis from 2013 is underpinned by several rationales. Firstly, while the Alipay account system's inception in 2004 could be construed as the genesis of digital finance in China, the industry commonly attributes the launch of Yu'EBao in 2013 as the seminal event marking the onset of digital finance development in the country [7]. Additionally, notwithstanding Bitcoin's genesis in 2008, scholarly research on Bitcoin and blockchain commenced gaining traction around the year 2013. Hence, this study adopts 2013 as the baseline for analysis.

It is imperative to acknowledge that the literature retrieval process was executed on February 26, 2023, and therefore, it is plausible that the database contents may have undergone alterations subsequent to this date.

The methodology for literature retrieval in this study was meticulously designed to comprehensively capture relevant scholarship within the domain of digital finance. This section also addresses the first research question: "RQ1. What are the main areas of research in digital finance?" Through defining the concept of digital finance and extensive review of relevant literature, the research topics covered in the field of digital finance include digital inclusive finance, fintech, internet finance, digital currency, cryptocurrency, blockchain, peer-to-peer lending, crowdfunding, mobile banking, mobile payments, insurtech, robo-advisors, and related derivative themes. The keywords for literature retrieval were determined based on the Chinese and similar expressions corresponding to these research topics.

Utilizing the CSSCI search criteria, the search terms were meticulously chosen to encapsulate various facets of digital finance:

SU = 'digital finance' + 'digital inclusive finance' + 'Fintech' + 'internet finance' + 'digital currency' + 'virtual currency' + 'Cryptocurrency' + 'Blockchain' + 'Bitcoin' + 'central bank digital currency' + 'CBDC' + 'decentralized finance' + 'DeFi' + 'peer-to-peer lending' + 'P2P' + 'digital bank' + 'Crowdfunding' + 'Mobile payment' + 'real-time payment' + 'Bigtech credit' + 'Insurtech' + 'Robo advisor'

At the same time, the search scope was limited to "Journal Article," with the subject classification set to "Economics and Management Sciences." No additional data filtering and cleaning were performed. This rigorous approach resulted in the identification of 5204 articles, providing a solid foundation for the subsequent scientometric analysis. This comprehensive literature search method aims to ensure a thorough examination of the digital finance research field, in line with the scientometric analysis principles outlined by Chen [20].

**Fig. 1:**
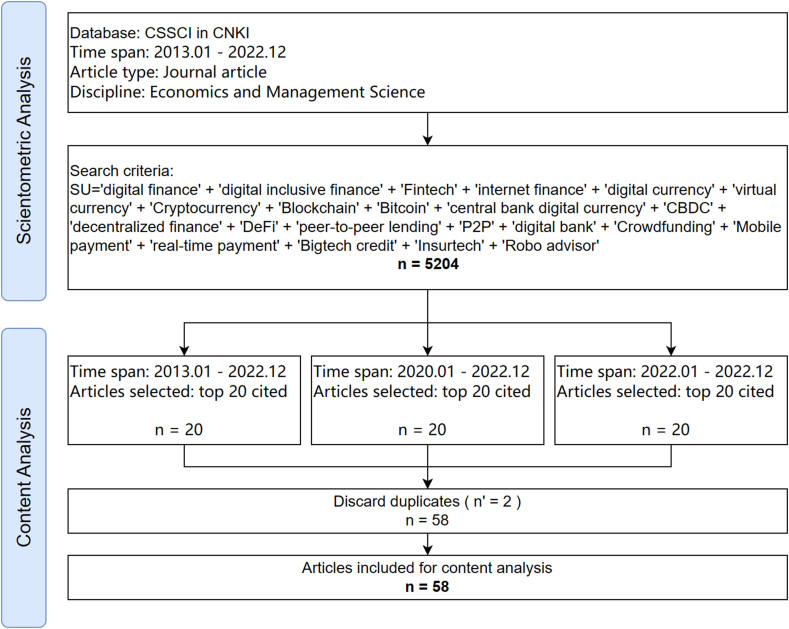
Flowchart of the literature data processing.

Scientometric analysis alone can struggle to provide detailed interpretations of article content. Therefore, to adequately interpret the research content in the field of digital finance in China, this paper supplements the initial literature data by selecting 58 of the most influential articles and combining them with key articles on relevant themes from the 5204-document dataset for content analysis. By setting the time frames to 10 years, 3 years, and 1 year, the 20 most cited articles within each period were chosen. After deduplication, this resulted in a subset of 58 articles. Through detailed analysis of these articles, including their abstracts, introductions, methods, results, and conclusions, this content analysis seeks to extract relevant insights and identify emerging trends. Fig. 1 illustrates the flowchart of the literature data processing.

## 3 Scientometric analysis

The primary objective of scientometric research is to conduct a systematic appraisal of scientific literature, thereby facilitating a comprehensive evaluation of the developmental trajectory of a particular subject across various dimensions, employing diverse indicators. Key facets of analysis encompass the historical evolution of publication quantity, distribution of publications across journals, author contribution distribution, and identification of highly cited articles, among others. These multifaceted analyses are facilitated through the utilization of Citespace software, leveraging the CSSCI literature data sourced from the CNKI database. This section will attempt to answer the second research question: "RQ2. In China, which institutions, journals, and authors are leading in the field of digital finance?" Additionally, it provides supplementary answers to the first research question: "RQ1. What are the main areas of research in digital finance?" within the context of the Chinese research environment.

Citespace software serves as a pivotal tool in this endeavor, enabling not only the classification and statistical analysis of literature data but also furnishing visual representations of the results gleaned from scientometric analysis. Through its functionalities, Citespace empowers researchers to discern overarching trends, delineate patterns of scholarly discourse, and elucidate the dynamics underpinning the evolution of a given research domain. By harnessing the capabilities of Citespace, scholars can derive actionable insights, inform decision-making processes, and contribute to the advancement of knowledge within their respective fields of inquiry.

### 3.1 Yearly Research Trend

**Fig. 2:**
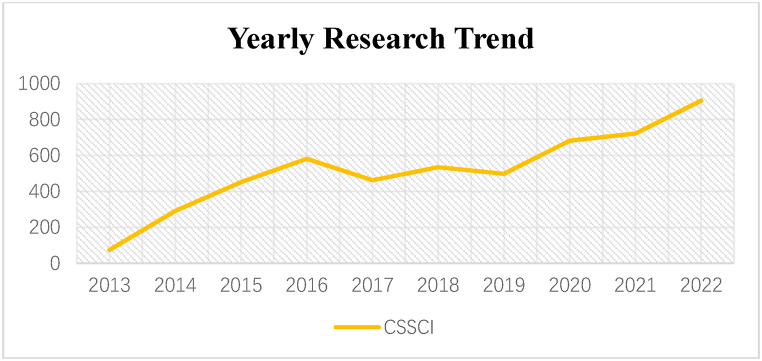
Yearly research trend.

After importing the filtered literature data into Citespace, the initial step involves categorizing and statistically analyzing the data based on the publication year. This process enables the determination of the number of scientific publications in each year. Table 1 illustrates the annual distribution of academic articles on digital finance research spanning from 2013 to 2022, while Fig. 2 offers a graphical depiction of the annual variation in publication numbers.

**Table 1:** Number of academic articles published per year.

Year	Count
2013	74
2014	291
2015	451
2016	581
2016	462
2018	535
2019	498
2020	682
2021	722
2022	905

From the discerned research trends in China, the evolution of digital finance research can be delineated into three distinct stages.

The first stage, spanning from 2013 to 2016, witnessed an explosive growth in digital finance research, propelled by the rapid proliferation of P2P lending in China since 2013.

The second stage, covering the period from 2017 to 2019, is characterized by relatively stable and moderate growth in the number of publications pertaining to digital finance research.

The third stage, comprising the years 2020–2022, witnessed a steady increase in the number of publications, indicative of a burgeoning phase of rapid development in digital finance research. The onset of this acceleration can be attributed to the global wave of digitalization catalyzed by the COVID-19 pandemic. In this context, digital finance assumes heightened significance as a vital component of the digital economy, thereby fostering a corresponding surge in scholarly research in this domain.

Overall, digital finance has emerged as a pivotal area of scholarly interest, with an overarching trend signaling sustained growth. The trajectory of future research within the realm of digital finance holds immense potential, promising continued exploration and innovation in this burgeoning field.

### 3.2 Research institution distribution characteristics

By extracting the C1 field from the data package, Citespace facilitates the analysis of the distribution of institutions to which the authors of the literature belong. Table 2 delineates the top 20 institutions with the highest number of scientific publications in the field of digital finance within CSSCI, based on the quantity of publications attributed to each institution.

**Table 2:** Most influential institutions.

Number	Institution	Count
1	Renmin University of China	216
2	Central University of Finance and Economics	158
3	Peking University	155
4	University of International Business and Economics	139
5	Nanjing University	111
6	Nankai University	98
7	Fudan University	90
8	Tsinghua University	88
9	Jilin University	88
10	Institute of Finance, Chinese Academy of Social Sciences	85
11	Southwestern University of Finance and Economics	83
12	Wuhan University	80
13	Shanghai University of Finance and Economics	78
14	Central South University	76
15	Zhongnan University of Economics and Law	66
16	Shandong University	66
17	Hunan University	63
18	Shanghai Jiao Tong University	57
19	Guangdong University of Finance	55
20	Xi'an Jiaotong University	55

The research findings underscore Renmin University of China's prominent position, leading significantly in terms of the number of published articles. It is closely followed by Central University of Finance and Economics, Peking University, and University of International Business and Economics. The distribution of remaining institutions generally follows a linear pattern.

It is noteworthy that the institutions of the authors exhibit a degree of concentration, with the top 20 institutions collectively contributing 1907 publications, representing 36.64 % of the total literature quantity. This concentration underscores the pivotal role played by a select group of academic institutions in shaping the discourse and advancement of digital finance research within the CSSCI domain.

### 3.3 Journal distribution characteristics

By extracting the SO field from the data package, Citespace enables the analysis of the distribution of source journals in the literature. Table 3 delineates the top 10 journals in terms of the number of publications within the field of digital finance in CSSCI.

**Table 3:** Most influential journals.

Number	Journal	Count
1	Journal of Financial Research	49
2	Exploration and Free Views	39
3	Management World	24
4	Guizhou Social Sciences	20
5	The Journal of Quantitative & Technical Economics	19
6	Journal of Social Sciences	19
7	Economic Research Journal	18
8	China Economic Quarterly	18
9	Science Research Management	18
10	China Industrial Economics	17

The cumulative number of publications from these top 10 journals amounts to 241, constituting only 4.63 % of the total quantity of literature selected from CSSCI. This observation suggests that the field of digital finance research lacks a core group of journals, with publication distribution being relatively dispersed.

However, it is noteworthy that the journals listed in Table 3 are all esteemed and highly regarded within the academic community, indicating their quality and prominence in the field. Nonetheless, the low proportion of their publication numbers also implies the existence of a substantial amount of low-quality literature disseminated across various non-prestigious journals within the realm of digital finance research in China.

This discrepancy underscores the need for Chinese scholars to exercise discernment and diligence in their publication endeavors. To enhance their influence within the field of digital finance, it is imperative for scholars to prioritize not only quantity but also the quality of their research output. By gravitating towards reputable and high-impact journals, scholars can amplify the visibility and impact of their contributions, thereby bolstering their standing within the academic community.

### 3.4 Core authors and author collaboration networks

According to the data extracted from the AU field of the data package, Citespace facilitates the extraction of author names from scientific literature. Table 4 presents the statistical data of core authors, listing the top 20 authors based on the number of publications attributed to each author.

**Table 4:** Most influential authors.

Number	Author	Count
1	Lu, Minfeng	21
2	Cheng, Xuejun	21
3	Feng, Sixian	20
4	Yang, Dong	18
5	Zeng, Hongjiang	18
6	Hu, Jinyan	18
7	Huang, Yiping	17
8	Zhuang, Lei	17
9	Pei, Ping	15
10	Liao, Li	14
11	Ba, Shusong	12
12	Yin, Zhichao	11
13	Zhou, Qin	11
14	Chen, Xiaohong	10
15	Zhang, Chenghu	9
16	Deng, Jianpeng	9
17	Shen, Yan	8
18	Liu, Zhiyang	8
19	Yin, Zhentao	8
20	Chen. Xiaohui	8

However, it is imperative to note that the number of publications alone may not accurately reflect the research caliber of authors. Therefore, in addition to considering publication volume, the total number of citations for the top 50 articles selected from CSSCI between 2013 and 2022 was compiled. By amalgamating the frequency of appearance and citation ranking of authors, we can discern the core authors in the field of digital finance.

Based on the comprehensive analysis conducted, three core authors have been identified in the realm of digital finance in China. They are Li Liao from Tsinghua University, Dong Yang from Renmin University of China, and Yiping Huang from Peking University. These scholars emerge as prominent figures within the domain, epitomizing excellence and substantial contributions to digital finance research in China.

**Fig. 3:**
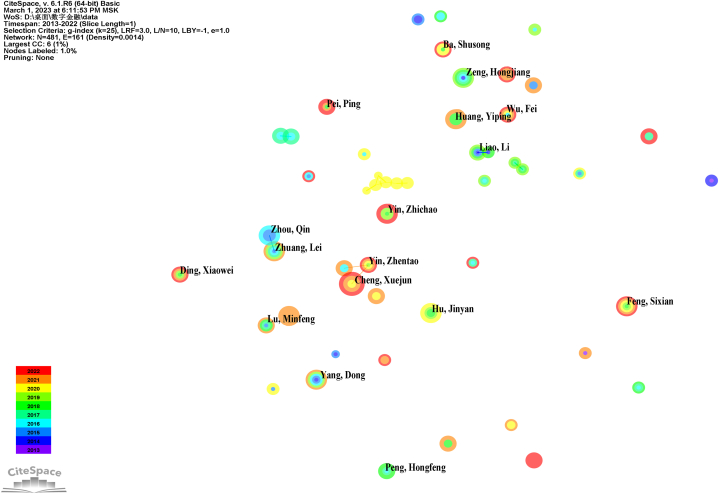
CSSCI Co-authorship network.

Fig. 3 illustrates the principal author collaboration network within CSSCI utilizing Citespace for visualization. The threshold utilized in this visualization is a publication count exceeding 15. Each year is represented by a corresponding color, as indicated in the bottom left corner of Fig. 3. The color of the node's annual ring denotes whether the author published articles in that specific year. The width of each annual ring within the node signifies the number of publications in the respective year, while the size of the node indicates the total number of publications by that author. The color of the links symbolizes the year of the first collaboration, while the thickness of the links denotes the frequency of collaborations between authors [20].

A notable observation from the visualization is the sparse connections between nodes, indicative of the absence of clustered author collaborations within the field of Chinese digital finance. However, when comparing this observation with the earlier institutional analysis, it becomes apparent that Chinese scholars are concentrated within specific institutions, yet their level of collaboration remains low. This disparity suggests a deficiency in effective collaboration among Chinese scholars, both within the same institution and across institutions and regions.

Within Chinese higher education institutions, author collaboration in CSSCI publications primarily revolves around relationships between doctoral or master's supervisors and their students, rather than among professors or senior scholars. This partial explanation underscores the inadequacy of Chinese scholars' influence in international journals. These findings underscore the imperative of promoting collaboration among professors or senior scholars in China to augment their influence within the field of digital finance. Efforts directed towards fostering robust collaborative networks among scholars hold promise for enhancing the visibility and impact of Chinese scholarship on the global stage.

### 3.5 Keyword distribution

To delve deeper into the current state of research in digital finance, this study extracted keywords appearing in CSSCI. Citespace selected keywords from the DE (Descriptor) field in the data exported from CNKI. Due to software limitations, a threshold of N = 50 was set, representing the top 50 keywords with the highest frequency in each time slice. Based on these parameters, the study extracted the top 20 keywords with the highest frequency in CSSCI, as presented in Table 5. Notably, the three keywords with the highest frequency in CSSCI are Financial Technology (468 occurrences), Blockchain (464 occurrences), and Digital Finance (354 occurrences).

**Table 5:** Top 20 keywords.

Number	Centrality	Year	Keyword	Count
1	0.1	2017	Fintech	468
2	0.13	2016	Blockchain	464
3	0.07	2018	Digital Finance	354
4	0.13	2013	Financial Regulation	207
5	0.05	2013	Digital Currency	165
6	0.18	2013	Commercial Bank	160
7	0.12	2014	Financial Inclusion	129
8	0.08	2013	Crowdfunding	127
9	0.03	2018	Digital Economy	126
10	0.06	2014	Financial Innovation	108
11	0.05	2014	Equity Crowdfunding	105
12	0.43	2013	Mobile Payment	105
13	0.07	2013	Big Data	98
14	0.09	2013	Bitcoin	92
15	0.01	2018	Financing Constraint	82
16	0.05	2014	Financial Risk	80
17	0.07	2013	Online Lending	79
18	0.05	2013	Monitoring	61
19	0.02	2018	RegTech	59
20	0.01	2019	Intermediary Effect	55

Table 5 further highlights the centrality of six keywords—Financial Technology, Blockchain, Financial Regulation, Commercial Banks, Inclusive Finance, and Mobile Payments—whose centrality values are 0.1, 0.13, 0.13, 0.18, 0.12, and 0.43, respectively. This indicates their significant bridging role across various research directions within the field of digital finance. Particularly noteworthy is the remarkably high centrality of Mobile Payments at 0.43, underscoring its pivotal core position in the realm of Chinese digital finance. Intermediate centrality, as proposed by American sociologist Linton Freeman [21], gauges the extent to which a node facilitates connections in the overall network by measuring the number of shortest paths passing through a node.

**Fig. 4:**
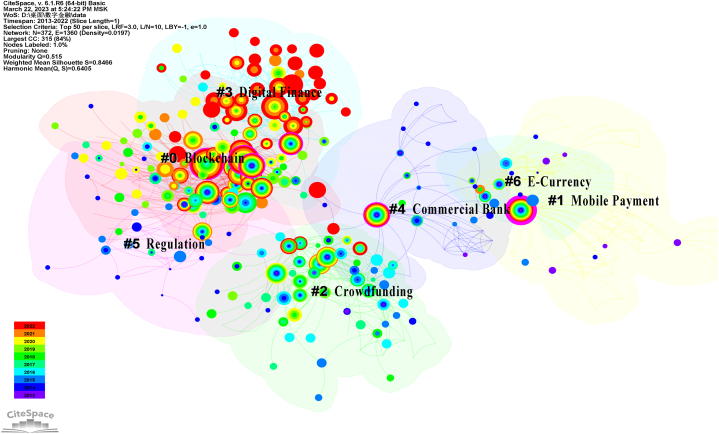
Visual mapping of CSSCI keyword cluster.

To comprehensively examine the knowledge structure of research in the field of digital finance, the Log-Likelihood Ratio (LLR) algorithm was employed to cluster closely related keywords. Table 6 furnishes detailed information about the clustering of CSSCI keywords, with each cluster containing a set of related keywords. Fig. 4 visually displays the largest and most significant seven keyword clusters in CSSCI, labeled as #0 Blockchain, #1 Mobile payment, #2 Crowdfunding, #3 Digital finance, #4 Commercial bank, #5 Regulation, and #6 E-currency.

**Table 6:** CSSCI keyword cluster.

ClusterID	Size	Silhouette	Year	Label (LLR)	Cluster
0	83	0.741	2016	Blockchain; Fintech; Financial regulation; Digital finance; Digital currency	Blockchain
1	59	0.932	2013	Mobile payment; Banking industry; Fintech; Digital finance; Financial sector	Mobile payment
2	58	0.85	2014	Crowdfunding; Equity crowdfunding; Online lending platform; Investor; Fintech	Crowdfunding
3	50	0.858	2018	Digital finance; Funding constraints; Intermediary effect; Blockchain; Inclusive finance	Digital finance
4	32	0.903	2014	Commercial bank; Yu'e Bao; Risk-taking; Financial disintermediation; Internet finance	Commercial bank
5	24	0.847	2014	Regulation; Internet finance; Innovation; Traditional finance; Development	Regulation
6	9	0.98	2013	E-currency; Monetary supply; Stored-value card; Mobile phone recharge card; Specific subject	E-currency

The labels assigned to the keyword clusters represent the research themes that have garnered attention in the field of digital finance from 2013 to 2022. This clustering analysis supplements the first research question: "RQ1. What are the main areas of research in digital finance?" within the context of the Chinese research environment, thereby providing important references for further exploration and inquiry in this field.

**Fig. 5:**
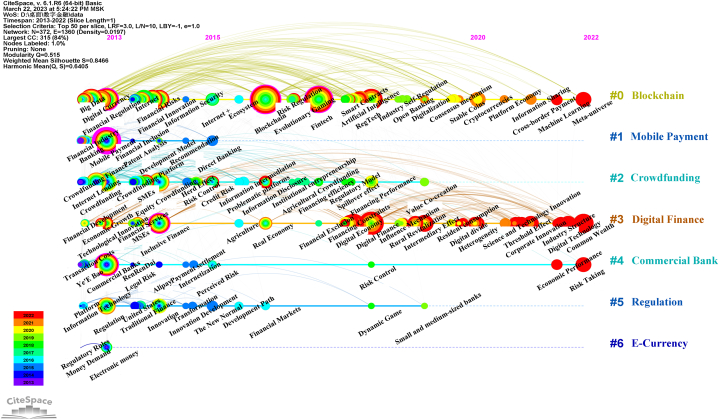
Visual mapping of CSSCI keyword cluster timeline view.

The timeline graph of keyword clusters, depicted in Fig. 5, has been rearranged based on the timeline to elucidate the developmental trajectory of each subfield within digital finance. In this graph, the position of each node denotes the time of the first occurrence of the corresponding keyword, while the size of the nodes is determined by the weighted results of centrality and frequency of occurrence. The color of the annual rings signifies the publication year of the corresponding keyword literature, with the width of the rings representing the number of publications.

Upon examining the timeline graph, two subfields, namely #0 Blockchain and #3 Digital finance, exhibit relatively complete and continuous development trajectories within Chinese digital finance research. It is noteworthy that the colors green and blue predominate in the node's tree rings of the figure, indicating that research across various subfields in China was primarily conducted before 2019. However, the colors yellow and red, representing the years 2020–2022, show a higher concentration of research in the #3 digital finance subfield.

To gain further insights into the research trajectory of the digital finance field, burst detection for keywords was conducted. Bursting keywords denote a significant increase in their frequency of use within a brief period, thereby representing research hotspots during specific time periods. In this study, a threshold of 2 years was set as the minimum occurrence year for burst detection.

**Fig. 6:**
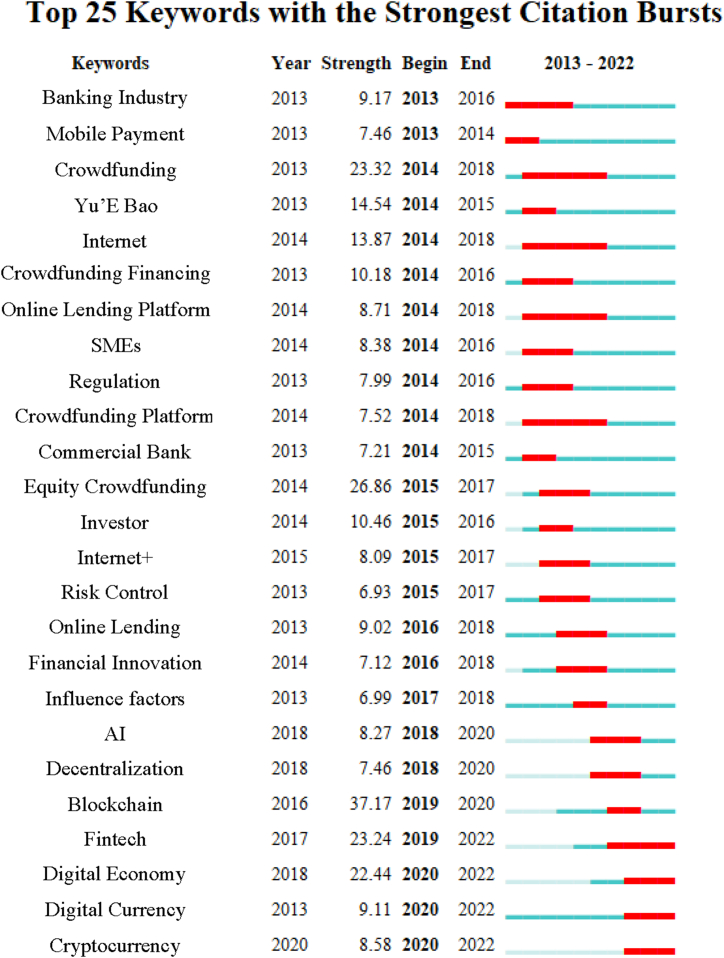
CSSCI Top25 keyword burst.

Fig. 6 illustrates the evolution of burst occurrences in CSSCI keywords, highlighting noticeable jumps and changes over time. Before 2018, research focus in the digital finance field was primarily centered around the mobile internet domain, encompassing areas such as mobile payments, online lending, and corresponding risk control and regulatory measures. However, with the commencement of China's P2P online lending industry clean-up in 2018, research in this domain underwent a significant shift.

Subsequently, research quickly transitioned towards international trends, focusing on the application of digital technologies such as blockchain, artificial intelligence, and cryptocurrencies. However, due to China's cautious stance towards cryptocurrencies, the progress of research in this area encountered obstacles, prompting divergent paths of exploration among Chinese and international scholars.

The development of the Peking University Digital Finance Index prompted Chinese scholars to pivot towards empirical research on the current state of digital finance development in China. Ongoing bursting keywords include financial technology, digital economy, digital currency, and cryptocurrencies, reflecting the continued exploration and dynamism within the field.

## 4 Content analysis

A detailed insight into the field of digital finance can help authors intuitively understand its evolution and research frontiers, identify research gaps, and propose future directions. In this section, we address the third research question: "RQ3. What are the most influential articles and themes in China, and what are the main research frontiers in the Chinese digital finance literature?" To answer this question, the study primarily analyzes the evolution of themes and research frontiers in the Chinese digital finance field from the perspective of literature citations.

Citations are widely recognized as a crucial means for researchers to establish their positions within their respective fields. By aligning with or opposing the work of other researchers and seeking opportunities to contribute new knowledge, citations serve as a key metric for evaluating scholarly impact and influence [22].

The selected articles in this study will be regarded as the cited literature, and their importance will be gauged based on the number of times they have been cited at the time of data extraction. This analysis aims to elucidate the distribution of the most significant articles in the field of digital finance in China across various research domains.

Through this approach, insights can be gleaned into the seminal works that have shaped and influenced the trajectory of research within the digital finance domain. Identifying these pivotal articles provides valuable context for understanding the prevailing discourse, trends, and research directions within the field.

### 4.1 Theme evolution analysis

We combined the keyword cluster (Fig. 4), the cluster timeline (Fig. 5), and the keyword burst (Fig. 6) from the scientometric analysis to examine the chronological trajectory of digital finance research in China. Clear trends emerge in the evolution of Chinese digital finance research.

The rise of mobile payments has been a major driver of rapid development in digital finance in China. It is noteworthy that cluster #6, labeled "E-currency," represents the electronification of money following the rise of mobile payments within the Chinese research context, rather than digital currencies such as CBDCs and cryptocurrencies. Subsequently, research in this area was gradually overtaken by studies focused on mobile payments. Research on crowdfunding and P2P lending, along with the regulatory challenges that emerged, ceased abruptly following the overall clearing of the P2P industry in China in 2018. Clearer main lines such as #0 blockchain, #3 digital finance, and #4 commercial banks have emerged, encompassing core themes such as fintech, digital inclusive finance, and CBDCs.

Table 7 highlights pioneering articles selected from CSSCI journals, depicting their significance from the perspective of citation scores. These scores are based on the total number of citations each article has received. The top 20 articles, included in the subset of 58 documents, are presented in the table. Pre-2018 investigations primarily revolved around internet finance and peer-to-peer (P2P) lending. Subsequent endeavors leaned towards empirical inquiries underpinned by the "Peking University Digital Finance Index" [9] as a foundation, alongside sustained attention to regulatory inquiries. It is noteworthy that the five most cited articles are from publications post-2018, all of which focus on digital inclusive finance as their research entry point. This marks the rapid development and sustained expansion of digital finance research in China in recent years.

**Table 7:** CSSCI most influential articles.

Number	Authors	Article Title	Source Title	Cited	Year
1	Guo, F; Wang, JY; Wang, F; Kong, T; Zhang, X; Cheng, ZY	Measuring China's Digital Financial Inclusion: Index Compilation and Spatial Characteristics	CHINA ECONOMIC QUARTERLY	2183	2020
2	Zhang, X; Wan, GH; Zhang, JJ; He, ZY	Digital Economy, Financial Inclusion,and Inclusive Growth	ECONOMIC RESEARCH JOURNAL	1786	2019
3	Xie, XL; Shen, Y; Zhang, HX; Guo, F	Can digital finance promote entrepreneurship? -- Evidence from China	CHINA ECONOMIC QUARTERLY	1333	2018
4	Huang, YP; Huang, Z	The Development of Digital Finance in China: Present and Future	CHINA ECONOMIC QUARTERLY	1271	2018
5	Yi, XJ; Zhou, L	Does Digital Financial Inclusion Significantly Influence Household Consumption? Evidence from Household Survey Data in China	JOURNAL OF FINANCIAL RESEARCH	1212	2018
6	Zheng, LS	China's Internet Finance: Model, Impact, Nature and Risks	INTERNATIONAL ECONOMIC REVIEW	1121	2014
7	Xie, P; Zou, CW; Liu, HE	The Necessity and key Principles of Regulation Over Internet Finance	STUDIES OF INTERNATIONAL FINANCE	1097	2014
8	Tang, S; Wu, XC; Zhu, J	Digital Finance and Enterprise Technology Innovation:Structural Feature, Mechanism Identification and Effect Difference under Financial Supervision	MANAGEMENT WORLD	1018	2020
9	Li, B; Dong, L	Models and developments in Internet finance	CHINA FINANCE	1011	2013
10	Wu, XQ	Internet Finance: The Logic of Growth	FINANCE & TRADE ECONIMICS	974	2015
11	Song, XL	Empirical Analysis of Digital Inclusive Finance Bridging the Urban-rural Residents' Income Gap	FINANCE & ECONOMICS	960	2017
12	Xie, P; Zou, CW; Liu, HE	The Fundamental Theory of Internet Finance	JOURNAL OF FINANCIAL RESEARCH	954	2015
13	Qiu, H; Huang, YP; Ji, Y	How does FinTech Development Affect Traditional Banking in China? The Perspective of Online Wealth Management Products	JOURNAL OF FINANCIAL RESEARCH	931	2018
14	Wang, HJ; Liao, L	Chinese P2P Platform's Credit Authentication Mechanism Research-- Evidence from Renrendai	CHINA INDUSTRIAL ECONOMICS	921	2014
15	Shen, Y; Guo, P	Internet Finance, Technology Spillover and Commercial Banks TFP	JOURNAL OF FINANCIAL RESEARCH	887	2015
16	Huang, HL	Research of the Internet Finance with the Core as E-commerce Platform	SHANGHAI FINANCE	808	2013
17	Wang, X	A Study on Internet Finance Helping Relieve SMEs Financing Constraints	JOURNAL OF FINANCIAL RESEARCH	747	2015
18	Liao, L; Li, MR; Wang, ZW	The Intelligent Investor: Not-Fully-Marketized Interest Rate and Risk Identify-- Evidence from P2P Lending	ECONOMIC RESEARCH JOURNAL	696	2014
19	Yang, D	Supervising and Regulating Science and Technology: Supervisory Challenges and Dimensional Construction of Financial Technology	SOCIAL SCIENCES IN CHINA	668	2018
20	Zheng, ZL	The Influence of Internet Finance of Commercial Banks-- Based on the Perspective of the Influence of “Internet +” on the Retail Industry	FINANCE & ECONOMICS	647	2015

Pre-2018, the focus of Chinese digital finance research predominantly centered on internet finance and peer-to-peer (P2P) lending, as evidenced by 10 pertinent articles cataloged in Table 7. Li & Dong [23] laid the foundation in 2013 by outlining three primary models of internet finance, with particular emphasis on P2P lending. The first model extended traditional financial services to the online realm, encompassing e-banking, online banking, and mobile banking. The second model encompassed internet intermediation services in finance, incorporating third-party payment platforms, P2P lending, and crowdfunding networks. The third model encapsulated internet financial services, encompassing platforms such as online small loan companies, internet funds, and insurance sales platforms. P2P lending in China experienced a meteoric rise from 2013 onwards, with significant expansion over the ensuing three years, culminating in a peak loan balance exceeding one trillion yuan by the close of 2017. However, regulatory interventions in 2018 led to a comprehensive shutdown of P2P lending platforms by mid-November 2020, halting its unsustainable growth trajectory.

Amidst the P2P lending boom, several pivotal studies emerged, shedding light on various facets of the industry. Wang & Liao [24] scrutinized data from "RenRenDai" to assess how its credit authentication mechanisms influenced lending behavior, while Liao et al. [25] delved into the micro-level factors shaping the marketization of interest rates, underscoring investors' adeptness in risk assessment and its role in underpinning interest rate marketization. However, the cessation of P2P lending spurred a shift in research focus towards other dimensions of internet finance. Huang [26] explored the genesis and contributing factors to the emergence of e-commerce finance, a significant subset of internet finance, categorizing it into consumer credit and loans for small and micro-enterprises facilitated through e-commerce platforms. Zheng [27] synthesized internet finance into four primary business models, encompassing financial institutions' information networking, third-party payment, online credit business, and virtual currencies.

Furthering the discourse, Xie et al. and Wu [28,29] provided comprehensive overviews of the theoretical underpinnings and growth trajectory of internet finance, emphasizing its transformative potential in enhancing financial inclusivity. Shen & Guo [30] expounded on the impact of internet finance on commercial banks, highlighting its role in augmenting the total factor productivity of Chinese commercial banks through technological spillover effects, albeit with varied impacts across different bank categories. Additionally, Zheng [31] juxtaposed internet finance with the retail industry, elucidating analogous pathways of influence and elucidating its profound repercussions on commercial banks' liability, intermediary, and asset businesses. Wang [32] unearthed the transformative impact of internet finance on the credit allocation landscape for small and micro-enterprises within traditional financial markets, advocating for a more rational allocation of financial resources.

As the digital finance landscape rapidly evolved, regulatory imperatives assumed paramount importance. Xie et al. [33] spearheaded the discourse by articulating the necessity and core principles underpinning the regulation of internet finance, emphasizing the pivotal role of prudential regulation, conduct regulation, and financial consumer protection. Subsequently, Yang [34] advocated for the incorporation of a technological dimension into financial regulatory frameworks, delineating the need for a dual-dimensional regulatory system to effectively address the risks inherent in financial technology and the attendant regulatory challenges it engenders.

Since 2018, the landscape of digital finance in China has stabilized, forming four research frontier themes with digital inclusive finance at the core.

### 4.2 Research frontier analysis

By combining content analysis with scientometric keyword analysis and excluding outdated themes during the evolution process, this paper ultimately identifies four main research trajectories in the Chinese digital finance literature: (1) digital currency; (2) digital inclusive finance; (3) fintech; and (4) blockchain technology. Each theme encapsulates a broad array of scholarly contributions and empirical investigations, reflective of the multifaceted nature of digital finance research.

This section will comprehensively analyze the subset of 58 influential articles, along with key articles from the 5204-document dataset on corresponding themes, to elucidate these thematic areas. The analysis aims to provide a thorough overview of the main research foci, pioneering literature, and emerging directions within the dynamic landscape of digital finance. Through this integrative approach, the study endeavors to furnish readers with nuanced insights into the evolving contours of scholarly inquiry in this burgeoning field.

(1) Digital Currency

The delineation of digital currency varies across global districts, encompassing a spectrum from cryptocurrencies to central bank digital currencies (CBDCs). This section aims to explore these two facets: private digital currencies and CBDCs.

Private digital currencies, exemplified by cryptocurrencies like Bitcoin, have undergone significant evolution since Satoshi Nakamoto introduced the concept in 2008. Notable cryptocurrencies such as Bitcoin, Ethereum, and Tether have dominated the market capitalization landscape. While international discourse on cryptocurrencies has flourished, scholarly investigations in China have predominantly focused on qualitative analyses, indicating a nascent research landscape in this domain.

In contrast, CBDCs have emerged as a pivotal response to the competitive landscape and challenges posed by private digital currencies. Unlike cryptocurrencies, CBDCs are issued or planned to be issued by central banks. Globally, numerous central banks are at varying stages of evaluating CBDC implementation. Noteworthy examples include the Sand Dollar by the Central Bank of The Bahamas, e-Naira by the Central Bank of Nigeria, and China's e-CNY, the first CBDC introduced by a major economy.

China's e-CNY, conceived within the Digital Currency/Electronic Payment (DC/EP) project plan initiated in 2015, operates on a two-layer framework. The central bank constitutes the first layer, with commercial banks, telecommunications operators, and third-party payment network platform companies forming the second layer. E-CNY has undergone extensive pilot projects and enjoys widespread adoption in China. The success of CBDCs hinges on their broad acceptance, with the digital Yuan potentially addressing entrenched issues within the conventional financial system [35].

(2) Digital Inclusive Finance

The proliferation of digital technology has revolutionized the efficacy of financial services, notably with the advent of mobile payment systems, which have substantially bolstered consumer convenience and emerged as pivotal constituents of digital financial innovation within the Chinese context. Beginning in 2013, platforms such as Yu'EBao and WeChat Pay have been introduced, catalyzing rapid adoption and advancement of mobile payment technologies across China. Additionally, digital technology has played a pivotal role in facilitating the provision of inclusive financial services, particularly catering to marginalized populations with limited incomes. A significant global contribution stemming from China's digital financial evolution has been the advancement of inclusive finance. Leveraging the expansive user base of Alipay, the Digital Finance Research Centre at Peking University, in collaboration with Alipay, has harnessed extensive micro-level data to devise the "Peking University Digital Inclusive Finance Index," encompassing 31 provinces, 337 cities at or above the prefecture level, and approximately 2800 counties in mainland China [9]. This index has laid the groundwork for further research endeavors in related domains. With the Peking University Digital Inclusive Finance Index serving as a pivotal explanatory variable, diverse empirical research avenues have emerged, solidifying China's preeminent standing in the realms of digital finance and inclusive finance research. Consequently, disparities in research focus have surfaced between Chinese and international scholars within this sphere. The ensuing analysis will scrutinize this subject matter from three distinct vantage points: macro, meso, and micro.

From a macroeconomic perspective, the ramifications of digital finance on the broader economic landscape are principally manifested in its implications for economic growth. Extant scholarship within China widely acknowledges the constructive role played by digital finance in fostering economic expansion and enhancing its qualitative aspects [36,37]. Investigations have delved into its contributory effects across various domains such as agriculture, rural development, regional innovation, and industrial structural refinement, affirming its catalyzing influence [[38], [39], [40]]. Significantly, the evolution of digital finance also facilitates inclusive growth within the Chinese economic milieu [41,42]. Another pivotal area of inquiry concerns the environmental ramifications of digital finance, particularly its impact on carbon emissions [43]. International scholarship predominantly gravitates towards this ecological aspect, substantiating the role and mechanisms through which digital finance contributes to the mitigation of carbon emissions [15], reduction of carbon intensity [44], and advancement of environmentally sustainable economic progress [14].

From a meso-level perspective, digital finance has introduced disruptions to the established economic geography and restructured the distribution of production factors to a certain extent. Concerning urban-rural income differentials, both theoretical frameworks and empirical analyses have corroborated the capacity of digital finance development to narrow the urban-rural income gap [[45], [46], [47]] and foster rural revitalization [48,49]. Regarding the objective of achieving common prosperity, extant literature presents divergent views. On one hand, certain studies affirm the poverty alleviation effects of digital finance [50] and its potential to advance common prosperity [51]. On the other hand, alternative scholars have identified that the expansion of digital finance heightens the likelihood of poverty and exacerbates multidimensional poverty, thereby engendering a discernible Matthew effect concerning common prosperity [52,53]. In terms of the labor force, the role of digital finance in facilitating labor mobility within China remains undisputed [54], as it promotes non-agricultural employment [55] and enhances the quality of employment opportunities [56].

From a microeconomic standpoint, the influence of digital finance extends to both individual income and consumption dynamics, as well as to the production and operations of firms. Concerning individuals, extant literature predominantly acknowledges the positive association between digital finance development and increased personal income [47,57], alongside its notable role in stimulating consumption patterns by mitigating liquidity constraints and streamlining payment processes [13,58,59]. For firms, the evolution of digital finance enhances the accessibility of financial services, particularly benefiting small and micro-enterprises and fostering entrepreneurship [60]. This advancement further catalyzes technological innovation within firms [[61], [62], [63]], notably in the realm of green innovation [63]. Additionally, research indicates a substitutive relationship between government subsidies and digital inclusive finance in driving firm innovation processes [64], underscoring its implications for informing governmental policy formulation and implementation in this domain.

(3) Fintech

The integration of digital technology with conventional financial institutions, including banks, securities firms, and insurance companies, is an ongoing process driving their evolution. A prevailing trend is the collaboration between technology firms and financial institutions, wherein technology companies offer technical solutions for financial services, while financial institutions harness digital technology to enhance the efficiency of their operations. This symbiotic relationship allows both parties to leverage their respective strengths and foster close partnerships. Thakor [65], in a comprehensive review of fintech literature, underscores the emphasis on the interplay between fintech and the banking sector. Extant studies have extensively examined the impact of fintech on banks from diverse angles, encompassing internet finance [66], bank risk management [67], credit provision [68], non-performing loan rates [69], and investment in banking services [12].

Technological advancement is recognized as a pivotal driver of total factor productivity growth, characterized by positive externalities and non-competitive attributes. Yang et al. [70] discerned that fintech enhances the total factor productivity of the banking sector through financial innovation, technology diffusion, and market competition, leading to substantial improvements in bank efficiency. Moreover, it has been established that fintech interventions can mitigate information asymmetry between financial institutions and enterprises, alleviate financing constraints, and optimize the allocation of credit resources, thereby augmenting the total factor productivity of enterprises [71]. By empowering traditional financial institutions with technology, fintech plays a crucial role in bolstering their ability to effectively serve the real economy.

(4) Blockchain Technology

Blockchain serves as an open distributed ledger capable of efficiently, verifiably, and permanently documenting transactions between parties [72]. Originally conceptualized by Satoshi Nakamoto in 2008 as the foundational technology supporting Bitcoin [73], blockchain has since found application across various domains, including numerous cryptocurrencies, smart contracts, supply chain management, and financial services.

One prominent application of blockchain technology is the metaverse, which delves into the creation of a persistent and decentralized online 3D virtual environment. The decentralized nature inherent to the metaverse gives rise to an independent operating economic system. Scholars have undertaken theoretical analyses to dissect the mechanisms, logic, and parallels with the real economy within the metaverse [74,75]. However, the realization of the envisioned metaverse faces hurdles due to technological limitations associated with the hardware devices and sensors necessary for real-time interaction within virtual environments, presenting significant implementation challenges.

In October 2021, the social networking conglomerate Facebook rebranded itself as Meta, with CEO Mark Zuckerberg articulating the company's dedication to metaverse development. Nevertheless, in February 2023, Zuckerberg announced Meta's departure from metaverse pursuits, redirecting its focus towards the field of artificial intelligence.

## 5 Discussion

This paper explores the development trends in digital finance and its supporting technologies. We also conducted a content analysis of four popular thematic clusters, highlighting the focus and interests of scholars and the academic community. Our findings provide insights for those committed to research in the digital finance field. Next, we address the final research question: "RQ4: What are the future research directions?"

Our analysis identified four main research themes: digital currency, digital inclusive finance, fintech, and blockchain technology. These themes reflect the current focus and trends in digital finance research in China. Compared to global trends, we see a similar emphasis on digital currency and fintech but a higher attention to digital inclusive finance, likely driven by China's policies aimed at poverty reduction and financial inclusion. This underscores the specific context of digital finance development and suggests that future research should consider regional policy frameworks when studying the impact of digital finance.

(1) Digital Currency

Our analysis indicates that the development and adoption of digital currency, particularly CBDCs, significantly impact the financial system. This supports the view that CBDCs can enhance payment efficiency and financial inclusion [76]. The rapid adoption and pilot projects of the digital Yuan also highlight unique challenges in the Chinese context, such as balancing innovation with regulatory control. Future research should explore how to address these challenges and what lessons other countries can learn from China's experience with implementing CBDCs.

(2) Digital Inclusive Finance

The rise of digital inclusive finance plays a crucial role in providing financial services to underserved populations. China's inherent advantages in digital finance, such as the extensive coverage of third-party payment platforms and rich data resources, have spurred a surge in empirical research on digital inclusive finance. The "Peking University Digital Inclusive Finance Index" has played a significant role in evaluating digital finance development through extensive micro-level data [9]. However, its applicability to international research is limited. National data security agreements restrict international scholars' access to data at the county level or below. Moreover, these data come from a single financial service provider and may not fully capture the entire scope of digital finance development in China. This limitation underscores the need to develop more comprehensive and globally accessible data sources and indices.

The current measurement methods and data limitations hinder the comprehensive adjustment and definition of digital finance research themes. These limitations impede research progress, indicating an urgent need to establish a unified theoretical framework and measurement standards in digital finance. These standards will help harmonize concepts and indicators, facilitating more coherent and comparative research across different contexts and regions.

At the macro level, integrating digital technologies into the real economy is crucial for high-quality economic development. Future research should explore strategies for more deeply and broadly integrating technologies like big data, cloud computing, artificial intelligence, and blockchain into various sectors, including manufacturing, agriculture, and services. This exploration should focus on these technologies' impact on productivity, industrial structure, and market competition, aiming to identify best practices and potential pitfalls.

At the meso level, the widespread application of digital technologies could widen the digital divide, exacerbating social inequality. Future research should investigate the impact of the digital divide on multidimensional poverty and explore policies to mitigate these effects. This includes studying how digital technologies affect the quality of life for impoverished groups in key areas such as education, healthcare, and finance, and proposing measures to ensure equitable access to and benefits from these technologies.

At the micro level, digital inclusive finance impacts different social groups differently. While it has been shown to increase income and access to financial services, the specific mechanisms affecting various demographics require further study. Future research should analyze how digital inclusive finance influences the economic behavior, consumption habits, and financial risk tolerance of different groups, such as urban and rural residents, different income levels, and genders. Understanding these nuances will help tailor financial products and policies to better meet the needs of diverse populations.

(3) Fintech

The integration of technology with traditional financial institutions has significantly improved efficiency and accessibility. Our review highlights that fintech helps traditional banks innovate and improve services, contributing positively to the development of the financial industry. This aligns with literature emphasizing fintech's role in enhancing the efficiency of financial services. While fintech's role in improving commercial banks' efficiency is recognized [71], its long-term impact requires deeper analysis. Future research should investigate the long-term effects of fintech on commercial banks' operational efficiency, risk management, and strategic transformation in various economic environments and policy contexts. Particular attention should be given to the application and improvement of fintech in commercial banking's assets and liabilities operations, providing insights for sustainable fintech integration.

(4) Blockchain Technology

Blockchain technology has the potential to revolutionize various aspects of the financial industry, including secure transactions, smart contracts, and decentralized finance. The development of the metaverse as a decentralized virtual environment is an example of the broader application of blockchain. However, technical limitations pose obstacles to realizing the envisioned metaverse. Future research should focus on overcoming these challenges and exploring the broader applications of blockchain technology, such as non-fungible token (NFT).

## 6 Conclusion

This study presents a comprehensive review of the literature on digital finance, utilizing the CNKI database for data extraction, yielding an initial pool of 5204 CSSCI papers. Following a meticulous selection process, 58 key papers were identified for content analysis, spanning the period from January 2013 to December 2022.

Initially, we used Citespace software to conduct a scientometric analysis, facilitating a visual representation of publication trends, major source journals, institutional and author collaborations, and keyword clustering. Subsequently, we performed a content analysis on selected key papers to describe the historical evolution, current status, popular themes, and frontiers of digital finance research in China. From this analysis, we identified four frontier research themes in digital finance: digital currency, digital inclusive finance, fintech, and blockchain technology.

Our study not only clearly defines the concept of digital finance and synthesizes the thematic scope of the field but also provides a Chinese perspective on research in this area. This study contributes to the literature by examining the development of digital finance in the Chinese context. It offers valuable insights into the specific challenges and opportunities brought about by digital finance from both theoretical and practical standpoints. For policymakers, our findings highlight the importance of supporting digital finance initiatives to promote economic development and financial inclusion. Financial institutions and technology companies can leverage these insights to drive innovation and improve service delivery.

It is imperative to recognize that the essence of digital finance lies in its financial underpinnings, propelled by continual advancements in financial functionalities, with digital technological progress serving as an enabling external condition for its evolution. China's rapid strides in digital technology and inclusive finance practices offer valuable insights and lessons for the international community.

Given the swift pace of digital technology iteration, particularly the exponential growth of AI technology based on LLM (large language model), the fusion of finance and technology is poised to persist, propelling the development and evolution of financial models at an accelerated pace and to a heightened degree.

This study serves as a valuable resource for young academic researchers, furnishing a clear understanding of the current state of digital finance research and offering insights for future scholarly pursuits. However, it is important to acknowledge several limitations inherent in this study. Firstly, the reliance solely on the CNKI database for literature extraction and the absence of co-citation analysis may constrain the comprehensiveness of the findings. This limitation stems from inherent constraints associated with the Citespace software utilized for scientometric analysis. Secondly, the data extraction process conducted by Citespace software may yield insufficient data nodes for each time slice, potentially hindering a comprehensive portrayal of analytical topics. Lastly, the content analysis, based on TGC frequency, may inadvertently overlook high-quality literature in niche research areas or recently published works, leading to a skewed representation of research hotspots and frontiers.

As emphasized earlier, research in the realm of digital finance is still nascent, and China's rapid advancements in digital technology and digital financial practices offer valuable insights for the global community to glean from and build upon.

### Data availability statement

Data is available upon request.

## CRediT authorship contribution statement

**Qiwei Li:** Writing – review & editing, Writing – original draft, Visualization, Validation, Supervision, Software, Resources, Project administration, Methodology, Investigation, Formal analysis, Data curation, Conceptualization. **Xinyu Zhang:** Writing – review & editing, Writing – original draft, Validation, Supervision, Software, Resources, Methodology, Formal analysis.

## Declaration of competing interest

The authors declare that they have no known competing financial interests or personal relationships that could have appeared to influence the work reported in this paper.
